# Real-world activities of clinician-researchers in perioperative medicine: an ethnographic qualitative case study embedded in a single-centre clinical trial

**DOI:** 10.1016/j.bjao.2026.100862

**Published:** 2026-09-15

**Authors:** Maria-Ximena Acero, Mathias Waelli, Mia Gisselbaek, Bernardo Bollen Pinto

**Affiliations:** 1 Healthcare Organisations Management Research Lab (HOM lab), Global Health Institute, Faculty of Medicine, University of Geneva, Geneva, Switzerland; 2 Arenes, CNRS, Inserm, EHESP, French School of Public Health, Rennes, France; 3 Division of Anaesthesiology, Department of Acute Care Medicine, Geneva University Hospitals, Geneva, Switzerland; 4 Unit of Development and Research in Medical Education (UDREM), Faculty of Medicine, University of Geneva, Geneva, Switzerland; 5 Department of Anaesthesiology, Pharmacology, Intensive Care and Emergency Medicine, Faculty of Medicine, University of Geneva, Geneva, Switzerland

**Keywords:** clinician-researcher, clinical trial, ethnography, patient engagement, perioperative medicine, perioperative research, work activities

## Abstract

**Background:**

Clinician-researchers play a key role in bridging clinical care and research, yet their day-to-day activities remain poorly described. This study aimed to characterise the range of activities undertaken by clinician-researchers in a perioperative clinical trial and explore how these activities are experienced by patient-participants.

**Methods:**

We conducted an ethnographic qualitative case study embedded in an RCT in a tertiary hospital. In phase 1, clinician-researchers (physicians and nurses) were observed *in situ* over 10 days using shadowing and time-motion analysis to document their activities. In phase 2, we conducted interviews with patient-participants. Data were analysed using an abductive approach informed by activity theory.

**Results:**

The study was conducted between October and November 2021 and included observation of seven clinician-researchers and eight patient-participants. Clinician-researchers engaged in a variety of activities extending past traditional research tasks. Time spent on supportive tasks was nearly three times as much as that spent on core RCT activities, with many tasks occurring before or after primary research procedures. Non-RCT activities accounted for one-third of observed time. Patient-participants reported that interactions with clinician-researchers contributed to their perioperative experience, particularly through (1) alleviation of boredom and (2) provision of a perceived sense of safety.

**Conclusions:**

Clinician-researchers undertook a wide range of activities that extended beyond protocol-driven research tasks encompassing significant coordination and relational work. These activities were perceived by patient-participants to positively influence their perioperative experience in this specific context. These findings highlight the importance of considering the full scope of clinician-researchers’ work when designing and implementing prospective clinical trials.

**Clinical trial registration:**

NCT04436016.

**Editor’s key points**

• In this single-centre study of real-world activities of clinician-researchers in perioperative medicine, the authors report that clinician-researchers spent less than one-quarter of their time on formal trial tasks, with much of their work involved in coordination, documentation, and trial preparation.
• Clinician-researchers involved in trials also provided patient care, emotional support, and guidance during hospitalisation.
• Patient-participants in the trial valued the regular contact and personal medical attention and described an added sense of safety during their perioperative care.

Amid growing concerns about workloads in healthcare organisations (HCOs), balancing research and clinical responsibilities has become a major source of conflict and a pressing managerial challenge.^1^ Clinicians engaged in research, including medical doctors, nurses, and physiotherapists, are referred to as ‘clinician-researchers’ and navigate dual roles.^2^^,^^3^ However, balancing research and clinical roles is complicated by several issues. First, owing to the shortage of healthcare professionals,^4^ clinicians dedicated to research projects often feel that their colleagues consider them ‘deserters’.^5^ Second, clinicians may perceive the research role as an ‘internal clash’^6^ with their primary mandate.^7^ The clinician’s mission prioritises the care of patients based on the principle of beneficence, whereas the scientist’s mission is centred on the pursuit of knowledge.^6^ Several studies have highlighted the ethical challenges faced by clinician-researchers, revealing the overlaps and potential contradictions between their research and clinical roles.^8^^,^^9^ Likewise, clinician-researchers may fear losing certain clinical skills owing to reduced exposure to clinical work.^10^ This dual role is thought to positively affect both HCOs and patient health outcomes.^11^^,^^12^ Therefore, numerous public initiatives worldwide promote these dual-career pathways for clinician-researchers.^13^, ^14^, ^15^, ^16^ However, there is a lack of data on how clinician-researchers’ research activities are defined, allocated, and implemented in specific contexts.^17^^,^^18^

Perioperative medicine, defined as multidisciplinary, patient-centred care for high-risk surgical patients during the perioperative period, is an evolving healthcare domain with broad potential.^19^ Optimal perioperative care relies on the collaboration between various healthcare professionals and medical specialties. Within this framework, ‘acute care’ clinician-researchers from anaesthesia and intensive care have emerged as key contributors to both perioperative care and research. Notably, their research of operating theatres or Intensive Care Units (ICUs). Therefore, the difference between the tasks officially included in the job descriptions of perioperative clinician-researchers from an acute care background and the activities they perform may be significant.

The primary aim of this study was to characterise the actual activities performed by perioperative clinician-researchers involved in the day-to-day conduct of a randomised clinical trial (RCT), including activities extending beyond protocol-defined research tasks. The secondary aim was to explore how patient-participants experienced these activities during their perioperative pathway.

## Methods

### Clinical research setting

The present study was embedded in the PROTECTIN-Pilot trial (Perioperative Cardioprotection With Ivabradine in Noncardiac Surgery),^20^ a double-blind, placebo-controlled feasibility RCT investigating the use of ivabradine *vs* placebo in high-risk surgical patients. The RCT was conducted at the Geneva University Hospitals (HUG) between October 2020 and January 2022. It was approved by the Geneva Research Ethics Committee (16 June, 2020, BASEC-2019-00424) and the Swiss Agency for Therapeutic Products (Swissmedic, 2020DR4100). The ethics committee application explicitly described the present qualitative study. All patient-participants provided written informed consent. In the patient information sheet, there was a specific reference to the present qualitative study, including descriptions of interviews with study participants and *in situ* observations during study visits. An oral consent for the qualitative study was further sought from a sample of participants immediately before interviews. The RCT was registered at ClinicalTrials.gov (17 June, 2020, NCT04436016). In summary, 78 patient-participants at high risk of cardiac complications after noncardiac surgery were randomised to a personalised regimen of ivabradine or placebo for 3 days, from the morning of surgery to postoperative day 2. Most study visits occurred in surgical wards where, in addition to routine care, the research team visited the patient to measure vital signs (three-lead ECG, HR, blood pressure, and peripheral oxygen saturation) and noninvasive cardiac output twice daily, from the morning of surgery until the morning of postoperative day 3. A 12-lead ECG was recorded before surgery and on the mornings of postoperative days 1–3 or when there was clinical suspicion of ischaemia or arrhythmia. Blood samples for cardiac biomarkers were taken before surgery and on the mornings of postoperative days 1–3.

At HUG, the perioperative care provided by anaesthetists is primarily limited to the preoperative assessment clinic, the PACU, and the perioperative intermediate care unit (IMC). Responsibility for postoperative care on the general wards lies with the surgical departments. Wards are organised by surgical specialty (e.g. digestive surgery, urology). Rooms accommodate one to two patients, and continuous vital sign monitoring is not available. Typical nurse-to-patient ratios in surgical wards range from 1:5 during the daytime to 1:10 overnight.

Anaesthetists from the IMC only become involved when patients experience severe pain, major complications, or deteriorating vital signs. Anaesthesia nurses clinical duties on the wards, except when participating in the acute pain team. Anaesthesia research nurses are part of the anaesthesia research unit and have no hierarchical relationship with the surgical departments.

### Qualitative ethnographic single-case study

In this study, we adopted an ethnographic approach inspired by the cultural historical activity theory (CHAT).^21^^,^^22^ CHAT is particularly well suited to documenting the actual work performed by physicians and nurses. The analysis focused on activities as situated, real-time practices within specific socio-material contexts,^7^^,^^23^ allowing us to capture tasks and interactions that may not be reflected in formal job descriptions.

The study comprised two complementary phases: (1) *in situ* observation of clinician-researchers’ activities during the day-to-day conduct of the trial and (2) semi-structured interviews with patient-participants to explore how these activities were experienced. This design observed practices and patient-reported experiences within a single empirical case.

The ethnographic single-case study approach allowed for an in-depth exploration of professional practices within their organisational context.^24^ Rather than aiming for statistical representativeness, the objective was to generate a contextualised understanding of how work is enacted in practice and identify mechanisms and patterns that may be transferable to similar settings.

From the outset, the analytical strategy combined structured coding of observed activities with abductive thematic analysis of interview data, enabling iterative movement among empirical observations, emerging categories, and existing analytical frameworks.

This study was conducted and reported in accordance with the Consolidated Criteria for Reporting Qualitative Research (COREQ) guideline. A completed COREQ checklist is presented in Supplementary Appendix 2.

The research team comprised two management researchers (M-XA and MW) and two medical doctors (BBP and MG), including two females (M-XA and MG) and two males (MW and BBP). No relationship was established with participants before the study. The team combined insider and outsider positions: the medical doctors were involved in the RCT and had direct knowledge of the clinical setting, whereas the management researchers were external to the clinical environment and had no clinical role. Observations were conducted by both clinician- and non-clinician-researchers, whereas interviews were conducted only by non-clinician-researchers, who were perceived by participants as external to the care team.

To ensure the rigour of our approach, we maintained reflexivity by holding team meetings and keeping a reflexive notebook during fieldwork, documenting assumptions and interests related to the topic. Collaborative coding and analysis by researchers with diverse professional backgrounds supported the iterative refinement of the analytical framework and enhanced the study’s rigour.

Interview transcripts and observational data are available upon reasonable request.

### Phase 1: *in situ* observation of clinician-researchers

#### Participants

The phase 1 population comprised all clinician-researchers involved in direct contact with patient-participants during the RCT data collection period. This approach aimed to capture the full range of activities performed within the study setting at a specific point in time.

#### Data collection

Three members of the research team (MX-A, MW, MG) conducted observations by shadowing,^25^ which involves closely following one clinician-researcher at a time and observing their activities and tasks. They took descriptive notes and discussed the objectives of their activities together. The shadowing was conducted in two stages: first, to allow familiarisation with the work environment and, then, to enable more focused observations. The initial fieldwork spanned 3 days, involved two medical doctors and one nurse, and aimed to create an analytical framework for clinician-researchers’ activities. In the second stage, the investigators observed all RCT team members for 10 days, recording the duration of each activity unit in accordance with the principles of time-motion studies.^26^ We defined activity units as collective actions with a shared purpose and unique relational form. The boundaries of these units were determined by the extent of clinician-researcher activity uninterrupted.^27^

#### Data analysis

1. Framework

To elaborate a framework for clinician-researcher activity, we drew on an existing analytical framework that distinguishes the nature (objectives and context) of activities performed by coordinators in healthcare settings.^27^ Adapting the framework followed an iterative process.^28^ After the initial observation period, the analytical process encompassed the following steps: (1) the development of an exhaustive list of the observed activities, (2) the construction of preliminary coding rules, (3) the independent coding of the same observation set by four researchers (M-XA, MW, MG, and BBP), and (4) iterative meetings to compare interpretations and resolve discrepancies through discussion until consensus was reached. These discussions informed the progressive refinement of the coding framework.

**Fig 1:**
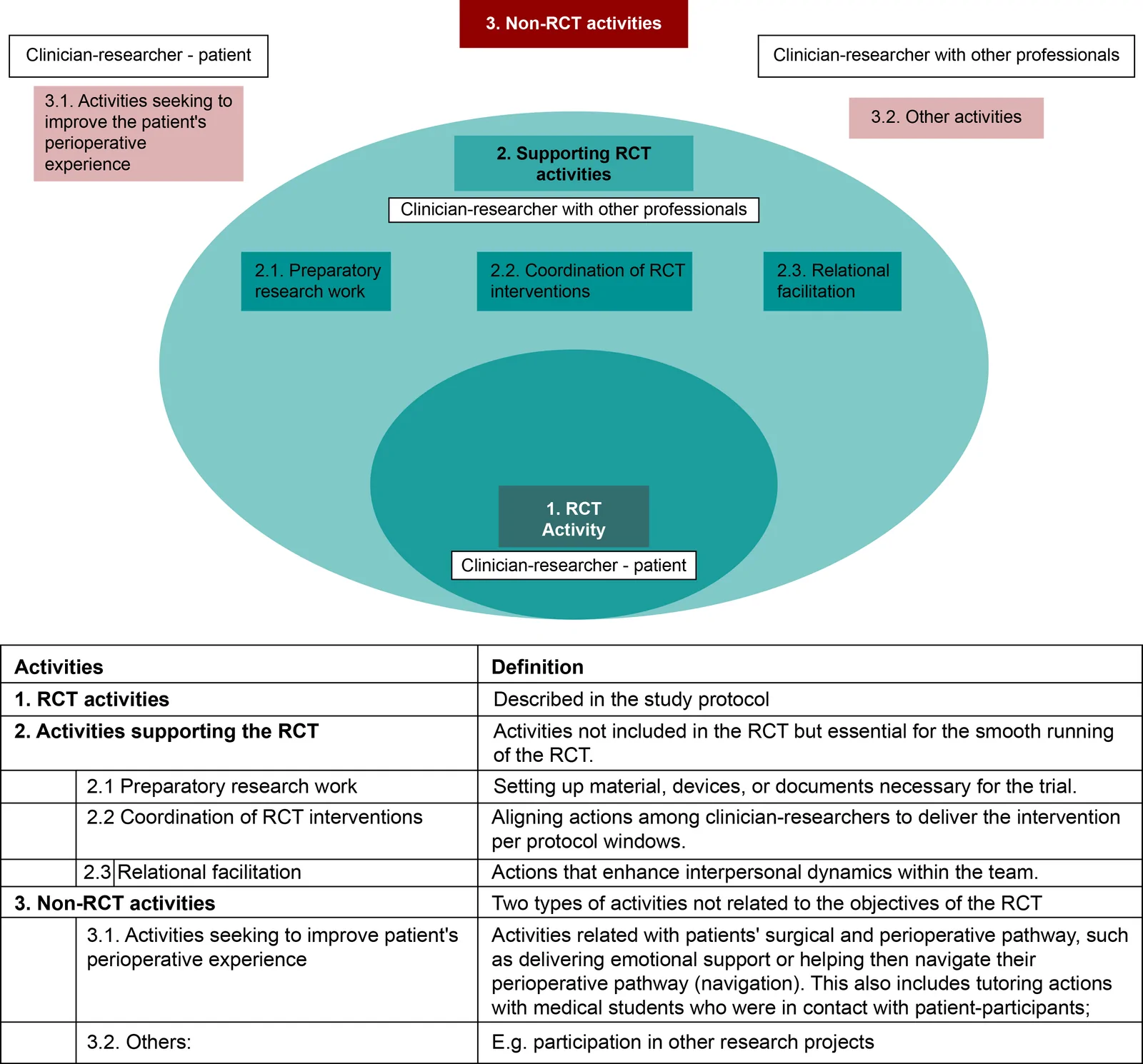
Framework adapted for the analysis of clinicians-researchers’ activity. RCT, Randomized Controlled Trial.

The final framework categorises the activities performed by clinician-researchers into three groups, as shown in Figure 1:

A) Activities performed as part of the RCT protocol
B) Activities that, although not specified in the protocol, contribute to the smooth delivery of the trial: (1) preparatory research work, (2) coordination of RCT interventions, and (3) relational facilitation
C) Activities that are unrelated to the RCT objectives: (1) enhancing the patient’s perioperative experience and (2) other research or clinical tasks

Each activity was also coded to indicate whether it involved patient-participants or other healthcare professionals.

2. Coding timed observations

M-XA and MW coded the activities for each clinician-researcher using the framework (Fig. 1) in spreadsheets (one spreadsheet per category) at HUG in October and November 2021. Then, data from nurses and doctors were compared with explore differences between the two professions and their respective types of organisations. Total observation time varied among clinician-researchers, and not all individuals engaged in activities within each of the three groups described in Fig. 1. To facilitate comparisons between nurses and doctors, activity times were normalised to each individual’s total observation time. For activities involving three or more clinician-researchers, data are presented as median percentages of total time with interquartile ranges. For activities with fewer than three clinician-researchers, individual-level data are presented.

### Phase 2: interviews with patient-participants

#### Participants

The phase 2 population comprised all patient-participants enrolled in the RCT who were still hospitalised and available at the end of phase 1, with no refusals. We used a convenience sampling approach, including all available patients who were likely to have been affected by the activities observed during phase 1.

The objective of the interview phase was not to achieve thematic saturation, but to capture patient-participants’ experiences within a bounded empirical context. Sampling adequacy was guided by the concept of ‘information power’, whereby the relevance of the sample depends on the specificity of the study aim, the homogeneity of the sample, and the richness of the data.^29^ This strategy aimed to provide a contextualised understanding of how the observed activities were experienced by patient-participants, thereby complementing the findings from phase 1.

#### Data collection

Semi-structured interviews (lasting approximately 30–50 min) were conducted and audio-recorded by M-XA and MW at HUG in October and November 2021. The interview guide encompassed three themes: (1) description of the interventions followed in the RCT, (2) motivation for participating in the RCT and in perioperative research in general, and (3) perceived impact of participation in the RCT on patient care and hospitalisation. The interview guide is presented in Supplementary Appendix 1. When asking patient-participants about their RCT journey, the interviewer highlighted the distinction between trial procedures and routine patient care.

The interviews were conducted in the surgical wards of the patient-participants to create a comfortable, open environment. Conversations often took a narrative turn as participants recounted their journey from the day of surgery to the interview date. The interviewer encouraged participants to provide specific details and examples relating to their unique experiences and needs at each stage of the RCT process.

#### Data analysis

The interviews were transcribed and analysed by M-XA and MW. The verbatim transcripts were then coded using thematic analysis with an abductive approach.^30^ The analysis involved iterative movement between the pre-existing categories reflected in the interview guide and the themes emerging from the data. For verbatim accounts that could not be classified, emerging categories were discussed in team meetings. Abductive reasoning emphasises the primacy of the empirical field, enabling researchers to maintain a critical distance from theoretical assumptions. The interviews were narrative and provided detailed accounts of the perioperative experience, enabling the identification of meaningful patterns in this specific context. Transcripts were not returned to participants, and no repeat interviews were conducted. The analysis was based on single interviews complemented by observations from phase 1, which contributed to the contextual interpretation of the data.

## Results

### Phase 1: *in situ* observation of clinician-researchers

The daily research activities of seven clinician-researchers were observed (see Table 1). These observations took place in surgical wards. In the first stage, three clinician-researchers (two doctors and one nurse) were shadowed for 3 days. Subsequently, the activities of all RCT team clinician-researchers were timed over a 10-day period. Among the observed clinicians, nurses had a defined proportion of their full-time equivalent dedicated to research activities, whereas only one physician had protected research time.

**Table 1:** Characteristics of the RCT clinician-researchers. All clinician-researchers were fully trained and board certified in their respective profession and specialty. FTE, full-time equivalent.

Clinician-researcher	Profession	Age	Sex	Clinical background	FTE % for research	Area of clinical work
RN-1	Nurse	46	Female	Paediatric intensive care	80	Paediatric intensive care
RN-2	Nurse	44	Male	Anaesthesiology	40	Anaesthesia
RN-3	Nurse	65	Male	Anaesthesiology	100	Anaesthesia
MD-4	Medical doctor	46	Female	Anaesthesiology and intensive care	0	Anaesthesia and perioperative care
MD-5	Medical doctor	39	Female	Anaesthesiology	0	Anaesthesia and perioperative care
MD-6	Medical doctor	34	Male	Anaesthesiology	0	Anaesthesia and perioperative care
MD-7	Medical doctor	42	Male	Anaesthesiology	50	Anaesthesia and perioperative care

#### Distribution of activities: RCT, support, and non-RCT work

In terms of work content, clinician-researchers engaged in activities unrelated to their RCT. Time spent performing RCT support activities (51 [15%] of the total observation time) was almost three times as long as time spent on the RCT activity itself (17 [12%] of the total observation time). These support activities were performed before or after the RCT activity. These activities involved administrative tasks such as collecting RCT data and documenting the case report form.

Coordinating actions between clinician-researchers, or between them and the patient’s care team, is another example of a support activity. Clinician-researchers used formal and informal means (such as RCT records, phone calls, e-mails, or scheduled or spontaneous meetings) to coordinate their activities. Clinician-researchers and the patient’s care team primarily coordinated through informal exchanges.

Non-RCT activities accounted for 26 (28%) of total observation time and included actions such as providing emotional support to patients and performing direct patient care tasks (e.g. adjusting oxygen therapy).

#### Differences between professional groups

Although patient-participants did not clearly distinguish between the roles of nurses and doctors, time-and-motion studies revealed differences in how the two groups allocated their research time.

**Fig 2:**
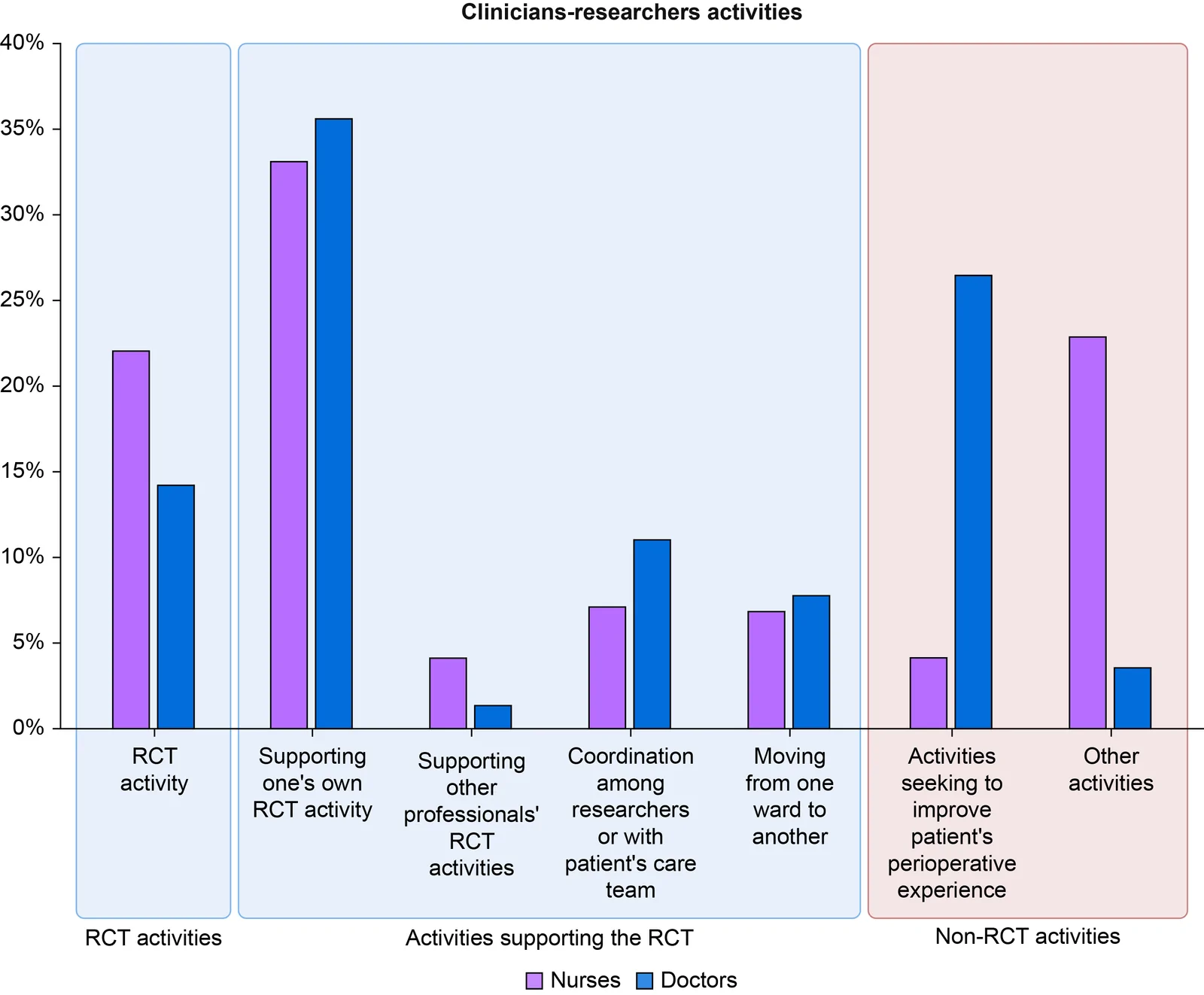
Comparative activity distribution between nurses and medical doctors. The distribution of activity was calculated as follows: time spent on each activity was expressed as a proportion of the total observation time.

Time dedicated to RCTs and RCT-supporting activities accounted for 70 (19%) of doctors’ time and 73 (14%) of nurses’ time (Fig. 2). RCT-specific activities represented 17 (17%) of doctors’ research time and 25 (16%) of nurses’ time. Doctors spent 11 (5%) of their time coordinating the work of other researchers and patient care teams, whereas nurses devoted 6 (11%) of their time to similar coordination tasks. In addition, one doctor and one nurse allocated part of their time to supporting RCT activities performed by other professionals, accounting for 5% and 9% of their observed time, respectively.

#### Engagement in non-RCT activities

Clinician-researchers were not exclusively involved in activities related to the RCT. Time spent on non-RCT activities accounted for 30 (40%) and 21 (24%) of doctors’ and nurses’ time, respectively. Non-RCT activities were divided into two sub-categories. First, activities aimed at improving the perioperative experience of patient-participants (including emotional support and assistance with navigating care pathways alongside RCT interventions) accounted for 4 (3%) of nurses’ time. Similar activities were also observed among two doctors, MD-5 and MD-7, accounting for 7% and 40% of their observed time, respectively. Importantly, clinician-researchers often integrated emotional support into routine research and clinical activities. For example, while preparing to measure vital signs, they engaged patient-participants in discussions about their hospital stay, perioperative pathway, or everyday life.

In addition, clinician-researchers helped the clinical teams on the ward to provide direct patient care. As part of the RCT, they collected patients’ vital signs and spoke to patients, thereby alleviating the clinical team’s workload.

Finally, other non-RCT activities accounted for 18 (25%) of nurses’ time. Most of this time was spent on research-related tasks associated with projects other than the observed RCT. Among doctors, only MD-5 was observed engaging in this category of activities, accounting for 23% of observed time.

### Phase 2: interviews with patient-participants

Between October and November 2021, we conducted interviews with eight patient-participants (10% of the 78 patients included in the RCT). The median age was 70 yr (7), and five participants were men. Most participants had an ASA physical status of 3 (*n*=6, 75%). Interviews were conducted between postoperative days 1 and 14 (Table 2).

**Table 2:** Characteristics of patient-participants. Further details are available in the main manuscript of the PROTECTIN-Pilot study.^20^ PS, SA physical status; D+, number of days after surgery.

Patient-participant	Sex	Age (yr)	ASA-PS	Surgery	Day of the interview
**1**	Female	74	3	Shoulder arthroplasty	D+3
**2**	Male	72	3	Colectomy	D+2
**3**	Male	66	3	Lower limb arterial endarterectomy	D+2
**4**	Male	78	3	Hip replacement	D+6
**5**	Male	73	3	Femoral-popliteal arterial bypass	D+6
**6**	Male	68	3	Cystectomy	D+14
**7**	Female	50	2	Lower anterior rectal resection	D+1
**8**	Female	68	2	Hip replacement	D+1

#### Perceived role of clinician-researchers across the perioperative pathway

Patient-participants did not express specific expectations regarding participation in the RCT. At most, they felt that they were not risking anything. Instead, their primary motivation was altruistic, driven by the desire to contribute to a research project that could potentially improve future patient care. However, in hindsight, the research had a significant impact on their hospitalisation experience after surgery. Patient-participants did not report perceived burdens or negative aspects associated with participation in the RCT. Their accounts predominantly described neutral or positive elements of their experience.

Patient-participants reported that clinician-researchers played a pivotal role in their care, overseeing their hospital journey from the preoperative anaesthesiology visit through the early postoperative period during hospitalisation and even after discharge (a telephone call was scheduled for 30 days after surgery). Operating across various units (preoperative assessment clinic, intensive/intermediate care, recovery room, and the surgical ward), clinician-researchers served as a consistent presence throughout the patient’s perioperative journey. Patient-participants portrayed clinician-researchers as a key resource during different stages of their perioperative pathway.

One patient-participant shared his experience with a clinician-researcher, stating, ‘He welcomed me the first morning of the surgery. And then this morning I saw him again. Finally, I had contact with clinician-researcher-3. It’s true that clinician-researcher-3 is a nice person... and I wasn’t butchered by him. I have no reason to be angry with him’ (interview, patient-participant 6).

The relationship developed between patient-participants and clinician-researchers was described as privileged, offering an improvement of their experience in two ways:

1. Breaking boredom

All patient-participants interviewed described their interaction with clinician-researchers as a welcome escape from the monotony of hospitalisation, as said by patient-participants 3 and 5 :

- ‘I saw several of your colleagues; the previous one I saw him in this room [...] we even talked about music a little bit’. (interview, patient-participant 3)
- ‘It didn’t bother me, on the contrary, it’s nice to have company’. (interview, patient-participant 5)
2. Perceived sense of safety

Four patient-participants expressed feeling reassured being followed by a third-party healthcare professional. For example, patient-participant 3 expressed:

- ‘I think I was even followed a little bit more by another method. If there had been any problem, it could be detected [...] I thought, well there’s an additional check’. (interview, patient-participant 3)

Patient-participant 6 also described this safety feeling and added:

- ‘We talked a lot. He took this role, I would say, himself. “How are you?” I told him, “I’m stressing a little, that’s it”. He immediately jumped up and told me the merits of this operation in a preventive way. It made me feel good to talk with him... Regardless of its role for study’. (interview, patient-participant 6)

## Discussion

Our study focused on a team of clinician-researchers comprising anaesthetist nurses and doctors. Combining ethnographic observations and patient interviews, we identified several key findings. First, we described a wide range of activities undertaken by clinician-researchers that went beyond those typically associated with conducting an RCT. For example, less than a quarter of clinician-researchers’ time was spent on RCT activities. They also dedicated time to coordinating RCT activities with patients’ care teams, enhancing the overall perioperative experience by providing emotional support and guidance on navigating care pathways and assisting clinical teams in delivering direct patient care. Second, patient-participants perceived participation in the RCT as positively influencing their perioperative experience by alleviating boredom and fostering a sense of security.

The differences between planned and real-world professional activity have been extensively studied in the sociology of work^31^^,^^32^ and activity theory^21^fields. Our observations in the context of a perioperative clinical trial confirm these findings: clinician-researchers engaged in numerous activities unrelated to the RCT, such as spending clinical time on tutoring/training activities and interacting with patients (providing emotional support or navigation). Previous research studying coordinating nurses also showed that more than 60% of their work was not directly related to their primary activity of coordinating patient care. In the context of emerging professions or roles with transversal functions, the literature has already demonstrated misalignment between prescribed and actual activities.^33^^,^^34^ The activities developed within these spaces, situated between prescribed work and actual work, represent an expression of subjectivity at work. This subjectivity responds to constraints arising from the local socio-material context and their identity dynamics.^23^ In our study, each actor adjusted their expected research activity in line with the constraints of their primary responsibilities. Some were fully dedicated to research, whereas others were primarily focused on clinical work. Hay-Smith and colleagues^35^ shed light on the multifaceted nature of the roles of clinicians who are also researchers, which extend beyond traditional research-related tasks. These roles encompass a variety of activities, such as collaborating with colleagues (referred to as ‘helping hands’), responding to patient-participant queries (termed ‘clinician requests’), and disseminating clinical insights gathered during the participant-clinician-researcher relationship. Our observations align with these findings, illustrating that the actual activities of clinician-researchers extend beyond the specific requirements of the RCT. Support activities aimed at facilitating the research project constituted a substantial part of their workload. Administrative duties also played a significant role, including preparing RCT records and meticulously documenting collected data. Both formal and informal coordination tasks were integral components of clinician-researchers’ activities. Finally, clinician-researchers spent part of their research time providing emotional support to patients.

In agreement with Hay-Smith and colleagues^35^ and Hiller and Vears,^36^ our study shows that patient-participants may not fully understand the dual roles of clinician-researchers. However, unlike Hay-Smith and colleagues,^35^ who reported that patient-participants had an ‘agenda’ when agreeing to participate in a clinical trial, those interviewed in our study did not indicate that they sought reassurance; rather, they said that simply knowing that their case was being monitored by another clinician (the clinician-researcher) made them feel safer. Other research, including that by Davies and colleagues,^37^ has raised concerns about potential patient-participants’ willingness to enrol in studies that pose a substantial risk of injury. Patient-participants in our RCT expressed altruism that motivated their participation. This difference could be explained by the fact that the potential benefits of the RCT intervention were valued more highly than the risks in the context of a preventive rather than a treatment- or ‘cure’-related intervention.

Our study also suggests that participating in an RCT may be associated with a more positive perioperative experience, as reported by patient-participants in this specific context. The engagement of clinician-researchers played a pivotal role in this regard. Previous studies have reported that patients experience boredom during hospitalisation, but patient-participants in the RCT said that interacting with clinician-researchers throughout their perioperative pathway alleviated feelings of boredom.^38^ Importantly, existing literature highlights the positive outcomes of clinician-researcher involvement in clinical research, particularly regarding relevance and research quality.^39^ Our study extends this understanding by suggesting that such involvement not only contributes to research-related aspects but also positively influences patients’ perceived comfort and safety during the perioperative period. This reported sense of safety may be related to actions performed during RCT, including clinical interventions such as ECG monitoring, taking vital signs, and adjusting oxygen therapy, and non-RCT activities such as providing emotional support and guiding patient-participants through their perioperative journey. Furthermore, active listening by clinician-researchers during the perioperative journey may influence patient-participants’ comfort and safety. However, because we did not include a control group of patients who did not participate in the RCT, the perceived increase in safety may be attributable to unmeasured factors not captured in our study.

The activities described in this study are specific to the operational conduct of an RCT embedded in routine clinical care. As such, the findings primarily reflect the work of clinician-researchers at the interface between research and clinical practice. Although some transversal dimensions identified in this study, such as coordination, relational work, and navigation across organisational boundaries, may be relevant to other forms of clinical research, their configuration is likely to differ in contexts involving other research phases (e.g. priority setting, governance, leadership) or methodologies (e.g. qualitative or non-interventional research). Further research is needed to explore how clinician-researchers’ activities vary across these different domains.

While acknowledging that clinical and research practices, healthcare funding models, and trial governance vary widely across global contexts, our primary objective was to explore how clinician-researchers’ roles and activities were distributed and their influence on the experiences of patients participating in a perioperative RCT. Our findings highlight the value that patients attribute to personal interaction, perceived oversight, and emotional support—elements that could enhance recruitment and retention if they were intentionally integrated into trial design. However, these potential impacts and their implications for trial design, funding decisions, and healthcare delivery were not directly examined in the present study. Such intentional efforts necessitate strategic planning, and healthcare leaders may be more inclined to fund clinical trials when informed of improved patient experience and the contribution of non-RCT activities by clinician-researchers to alleviating the burden on the healthcare system.

### Strengths and limitations

The present study has limitations. First, although we observed all the clinician-researchers available on the RCT team, this encompassed only a small number of individuals. The findings are shaped by the specific institutional, organisational, and interpersonal contexts of this setting and may not be generalisable. Heterogeneity in the activities observed among clinician-researchers was reflected by the fact that some individuals did not perform activities within all three groups. Within each professional category (medical doctors and nurses), participants were broadly comparable in seniority. Although one doctor held additional trial management responsibilities, the activities observed in this study—patient interactions during routine study visits—were consistent across participants. Therefore, seniority and role were not considered to have significantly influenced the findings. However, the use of abductive reasoning and thematic analysis enabled the development of conceptual categories and an analytical framework that may be relevant beyond this specific case. In line with Drisko,^40^ such transferability relies on readers’ ability to recognise meaningful similarities between the study context and other settings, thereby allowing the thoughtful application of findings to comparable situations. In addition, the small sample size of patient-participants (*n*=8) limits the breadth of perspectives represented and may have constrained the emergence of broader themes, reducing the transferability of findings. A future multi-site study involving other clinician-researcher teams could enhance the external validity and transferability of our findings. To increase internal validity, a larger cohort could be used.

The perspectives of the direct care team were not explored through interviews. Although interactions between clinician-researchers and care teams were observed, the perceptions of surgical staff and ward nurses were not directly captured.

Second, the nature of interviews with patient-participants introduces the possibility of interviewer bias. Although an interview guide was used to ensure consistency, the researchers’ previous assumptions or experiences may have influenced their interpretation of participants’ responses. Reflexivity was used throughout the analytical process to mitigate this, but the complete elimination of bias is impossible. All interviewed participants had agreed to take part in the RCT, which may introduce a selection bias. These individuals may differ from patients who declined participation in the RCT, particularly in their attitudes towards research, trust in healthcare professionals, or expectations of care. This may have contributed to the predominantly positive perceptions reported in this study. However, exposing patients who are not participating in an RCT to research activities conducted by clinician-researchers would be contrived and infeasible.

Third, potential harms or burdens of trial participation were not explored through a dedicated interview question. Although participants did not spontaneously report negative effects, some adverse experiences may therefore have remained silent. In addition, interviews with clinician-researchers were not conducted. Although such data could have provided additional insight into professionals’ perspectives, our methodological approach prioritised direct observation of work practices and their interpretation from the patient perspective. Future research could incorporate clinician-researchers’ accounts to further explore how these practices are understood and negotiated by the professionals themselves.

Finally, the inherently subjective nature of qualitative analysis means that there may be alternative interpretations of the data. Triangulation and peer debriefing were used to enhance the rigour of the analysis, but corroboration and extension of these findings would require further research in varied settings.

Our study has several strengths. Research into strategies that enhance the perceived value of research activities, such as those performed by clinician-researchers, could strengthen engagement with perioperative studies and improve trial participation. Engaging patients and participants in anaesthesia and perioperative trials remains challenging, partly owing to the public’s lack of awareness of the importance of perioperative complications, but also because anaesthesiologists have limited expertise in the emerging field of patient and public involvement in research. Our detailed analyses of the activities actually performed by clinician-researchers could also persuade managers and human resources teams to recruit more healthcare professionals for research roles and to oversee multidisciplinary teams within academic healthcare institutions. Furthermore, the positive perception of clinician-researchers among patient-participants may promote their role within the healthcare workforce.

### Conclusion

Our results highlight the diverse and vital roles of clinician-researchers in shaping perioperative pathways. These contributions were perceived by patient-participants as positively influencing their perioperative experience within this specific context and fostering improved collaboration within care teams. Furthermore, by integrating research with clinical practice, clinician-researchers contribute to the development of evidence-based protocols that can improve patient outcomes.

These findings highlight the multifaceted roles of clinician-researchers and suggest that their presence may be perceived by patient-participants as positively influencing their perioperative experience. Although the findings may be relevant to the management and coordination of clinician-researcher teams, these implications were not directly investigated in the present study and should be explored in future research. In particular, patients’ perception of increased safety emerged as a significant, potentially impactful finding that clinician-researchers could leverage strategically to improve future recruitment efforts. Conversely, mitigating patient boredom, which was also identified as a benefit, could be achieved through other means, suggesting that this function could be delegated in future models. The potential economic implications of our findings for health services, such as improved efficiency, reduced patient anxiety, and enhanced staff coordination, merit further investigation. Future studies should investigate how these perceived and operational benefits translate into economic value for healthcare systems. Furthermore, future research should explore the activities of clinician-researchers and their impact on patient-participants in other contexts. Qualitative research into patients’ expectations and barriers could inform strategies to improve participation in perioperative trials.

## Authors’ contribution

Study conception: MW, BBP

Study design: M-XA, MW, BBP

Data acquisition: M-XA, MW, MG

Data analysis: M-XA, MW

Data interpretation: M-XA, MW, MG, BBP

Drafting of the manuscript: M-XA, MW, MG, BBP

Revision of the manuscript: all authors

Approval of the final manuscript version: all authors

## Funding

‘Young Researchers Award’ (PRD 15-2018-I); Institutional funding from the Geneva University Hospitals.

## Declaration of interest

The authors declare that they have no conflicts of interest.
