# Supplementary material for: Real-world activities of clinician-researchers in perioperative medicine: an ethnographic qualitative case study embedded in a single-centre clinical trial
Source: BJA Open. 2026 Sep 15;20:100862. doi: 10.1016/j.bjao.2026.100862 (PMC13595070; doi:10.1016/j.bjao.2026.100862)
Supplement: Supplementary file 1 [file mmc1.docx]

**What do clinician researchers really do? A qualitative case study embedded in a perioperative clinical trial.**

**Appendix S1. Semi-structured interview with patient-participants.**

Présentation

- Objectif général de l’entretien.
- Rappel des principes de confidentialité et d’anonymat.
- Consentement verbal du patient (participation et enregistrement).

**Introduction**

1. Pourriez-vous me parler brièvement de vous ? (par exemple : âge, profession, parcours médical, etc.)
   - Depuis combien de temps êtes-vous hospitalisé ?
I. **Expérience des interventions RCT**
2. Pouvez-vous décrire votre participation dans l’étude ? Quand a-t-elle commencé ? Quelles personnes avez-vous vues ?
   - Consultation d’anesthésie ?
   - Appel téléphonique ?
   - Séjour à l’hôpital ?
3. Lors de votre séjour à l’hôpital, qu’est-ce que vous avez observé ou ressenti lors des interventions réalisées par les chercheurs ? Pouvez-vous les différencier de celles réalisés par l’équipe soignant ?
   - Pouvez-vous me donner des exemples ?
   - Quelles informations vous avaient été communiquées à propos de ces interventions ?
II. **Perception de l’impact**
4. Pouvez-vous me donner un exemple concret d’une situation qui vous a marqué(e) ?
   - Au-delà des aspects purement médicaux, y a-t-il d’autres changements que vous associez à votre participation ?
5. Quelles différences, le cas échéant, avez-vous pu identifier par rapport à votre parcours habituel ?

**III. Motivations pour la participation à l’étude**

6. Avez-vous déjà participé à d’autres études ou essais cliniques auparavant ?
7. Qu’est-ce qui vous a motivé(e) à participer à l’étude PROTECTIN ? Avez-vous discuté de votre participation avec votre médecin, votre famille ou vos proches ? Comment ces échanges ont-ils influencé votre choix ?
